# Added Value of Meat Inspection Data for Monitoring of Dairy Cattle Health in the Netherlands

**DOI:** 10.3389/fvets.2021.661459

**Published:** 2021-07-15

**Authors:** Anouk M. B. Veldhuis, Debora Smits, Martijn Bouwknegt, Heleen Worm, Gerdien van Schaik

**Affiliations:** ^1^Royal GD, Deventer, Netherlands; ^2^Vion, Boxtel, Netherlands; ^3^Department of Population Health Sciences, Faculty of Veterinary Medicine, Utrecht University, Utrecht, Netherlands

**Keywords:** meat inspection, surveillance, cattle, health, trend analysis

## Abstract

Meat inspection records of one large cattle slaughterhouse were analyzed to evaluate the added value of slaughterhouse data for cattle health surveillance in the Netherlands. Data were available from January 2015 to September 2018, consisting of 467,361 meat inspection records. Analyses included (1) an assessment of the representativeness of the cattle herds in the slaughterhouse data in relation to the cattle herd population in the Netherlands, and (2) multivariable analyses to quantify associations between meat inspection findings and farm of origin characteristics, and the trends in time of the findings in slaughtered cattle. Ninety percent of the meat inspection records originated from dairy cattle therefore this paper only presents the results of dairy herds (*N* = 422,194 cattle). The dairy herds in the slaughterhouse data seemed representative for the Dutch dairy population although their regional coverage differed from the distribution of dairy herds in the Netherlands. Non-dairy herds were underrepresented in the slaughterhouse data which stresses the importance of the inclusion of data from other slaughterhouses that may be more specialized in slaughtering beef cattle. Inspection records were categorized into 15 indicators related to ante-mortem and post-mortem findings. Following multivariable analyses, seven indicators were deemed of added value to existing cattle health surveillance components, as they provided either new information or information regarding specific health problems.

## Introduction

Since 2002, a national cattle health surveillance system is in place in the Netherlands that consists of, amongst other surveillance components, a trend analysis surveillance component (“TASC”) to monitor trends and developments in cattle health using routine census data (1). Briefly, stakeholders are informed on trends in key monitoring indicators such as mortality, fertility and udder health based on quarterly analyses of census data sources. When deemed relevant, additional in-depth analysis are performed to improve the models or to explore the potential of new data sources that could capture indicators of cattle health. The current study was carried out to assess the added value of meat inspection data in this context.

Cattle sent to slaughter undergo ante-mortem (AM) and post-mortem (PM) inspection by an official veterinarian or auxiliary meat inspector, to detect lesions that represent food-borne zoonotic infections. For example, PM meat inspection provides an important mechanism for detecting bovine tuberculosis (bTB) infections in cattle herds through the detection of bTB-like granulomas (2). In addition, meat inspection enables sentinel surveillance for animal health and welfare issues for which clinical surveillance is of limited sensitivity, such as foot and leg disorders and liver fluke infections (3). Given the systematic collection, its pre-diagnostic nature and large coverage, population-level meat inspection data has the potential to be a source of meaningful animal health information. Previous studies on slaughterhouse data revealed that certain characteristics of slaughtered cattle, such as sex and age of the animal and mortality rate in the herd of origin, are risk factors for partial, or whole carcass condemnation (4, 5). Analyzing data of condemned cattle carcasses could therefore be used to inform a risk-based surveillance approach of cattle health. Besides condemnation, changes in the trend of more specific AM- and/or PM-findings could reflect the occurrence of health disorders in the wider cattle population.

To evaluate the added value of slaughterhouse data for the cattle trend analysis surveillance component in the Netherlands, inspection results of one large cattle slaughterhouse were analyzed in this study. The study objective was 2-fold: (a) to assess whether the study population in the slaughterhouse data was representative for the target population (i.e., dairy and non-dairy herds in the Netherlands), and (b) to assess whether the trend in meat inspection findings and their association with characteristics of the farm of origin yields relevant input for the monitoring of trends in cattle health.

## Materials and Methods

### Meat Inspection Data

In the Netherlands, up to 650,000 adult cattle and over 1.5 million veal calves were sent to slaughter per year between 2015 and 2018 (6). Veal calves are mainly slaughtered in specialized slaughterhouses. A dataset with demographic and health related data of 467,361 adult cattle originating from Dutch farms and slaughtered between January 1, 2015 and September 30, 2018 was available from a cattle slaughterhouse located in the south of the Netherlands. These animals undergo AM- and PM inspection by official veterinarians or auxiliary meat inspectors, i.e., employees of an independent external organization (referred to as “meat inspection” from this point forward). The meat inspection is performed according to the specific rules for official controls on products of animal origin laid down in Regulation (EC) 854/2004 of the European Parliament. The dataset comprised herd of origin, animal identification, sex, age, signs observed during AM and PM inspection and reasons for condemnation of each animal. Herd and animal identification numbers were anonymised by an external enterprise prior to analyses.

### Data Analysis

#### Validation of Representativeness

For each herd in the dataset, the number of slaughtered cattle was aggregated by quarter of the year. These data were then merged with other routinely collected datasets containing herd type (dairy/non-dairy), herd size, region, on-farm cattle movements, and herd health certification statuses. These datasets were made available by nationally operating data collecting organizations and comprised the whole cattle population in the Netherlands. All data were anonymised by an external enterprise prior to analysis. More details on these data can be found in (1). The aforementioned herd characteristics were compared between the target population and the study population to assess the representativeness of the study population. About 90% of the meat inspection records originated from dairy herds. This paper therefore only presents the results of dairy herds.

#### Classification of Meat Inspection Findings

During the study period, 53 unique AM-findings and 79 unique PM-findings were recorded. A full list of the AM- and PM-findings is available upon request from the corresponding author. To identify meaningful trends and associations, AM-findings and PM-findings were categorized in 22 AM- and PM-categories using expert consultation. The team of experts consisted of a cattle veterinarian, a zootechnical specialist, an employer of the slaughterhouse and an employer of the competent authority responsible for the meat inspection in the slaughterhouse. “No AM-findings” and “No PM-findings” were added as additional categories as they potentially represent favorable animal health.

#### Multivariable Analyses

Statistical analyses were performed using STATA/SE version 15.1 software. For each herd, the number of slaughtered cattle with a finding in a specific category *i* was calculated per quarter *t*. Multivariable analyses where then conducted to quantify associations between characteristics of the herd of origin (i.e., the explanatory variables) and the herd-level frequency of AM- and PM-findings of each category of findings. Characteristics of the farm of origin were based on routinely collected census-data and included herd size, region, herd health certification statuses for endemic diseases [Salmonellosis, Leptospirosis, Bovine Viral Diarrhea Virus (BVDV), Bovine Herpes Virus-1 (BHV-1), and Para tuberculosis], antibiotic usage, annual replacement rate, farming system (open/closed), standardized milk production level (expressed as mean yearly net revenue per cow per herd), and average age of the slaughtered cattle (Table 1). More details on these data are described by Santman-Berends et al. (1). Slaughter cow prices, replacement cow prices and milk prices were retrieved from (7) and included as national averages per quarter of the year. Season, milk- and feed prices and quarter of the year were forced in the model as potential confounders. Independent continuous variables were categorized into four categories (10% smallest, 40% smaller, 40% larger, and 10% largest). For the independent categorical variables, the mean of the whole study population was included as the reference category). A population averaged panel-data model (xtgee) was fitted on each category of findings (i.e., the dependent variables) using a negative binomial distribution, a log link function, the unique herd identifier as panel variable, the year-quarter as time variable, the number of slaughtered cattle per herd per quarter as exposure variable and an independent correlation structure, in accordance with existing models of animal health indicators in the TASC (1). The model can be formulated as:

(1) $$\begin{array}{l} \ln\left(y_{it}\right)=\mu_{t}+\beta_{1}X_{1it}+\ldots +\beta_{n}X_{nit}+\varepsilon_{it} \end{array}$$

Where:

ln(*y*_*it*_) = natural logarithm of the number of cattle with a finding of category *x* in herd *i* in quarter *t*
μ_*t*_ = intercept for quarter *t*
_β_1, …*n*_*X*1, …*n it*_ = independent variable term for herd *i* in quarter *t*, for independent variables 1,…*n* as described in Table 1.
ε_*it*_ = random error for herd *i* in quarter *t*

**Table 1:** Characteristics of dairy herds in the slaughterhouse dataset that were used as independent variables in the multivariable analyses to quantify associations between herd characteristics and the herd-level frequency of AM- and PM-findings amongst slaughtered cattle in one Dutch slaughterhouse (*N* = 10,406 dairy herds).

**Characteristic**	**Category**	**Mean/Frequency**
Quarter-year	1–15	n.a.
Age at slaughter (months)	Continuous	65.3
Antibiotic use in cattle 1–2 years of age (% of herds)	No	86%
	Yes	14%
Antibiotic use in cattle >2 years of age in mean Defined Daily Dose Animal (DDDA)	10% herds with lowest DDDA	0.32
	40% herds with lower DDDA	1.81
	40% herds with higher DDDA	3.55
	10% herds with highest DDDA	5.62
BHV-1 status (% of herds)	Free	38.3%
	Non-free or unknown	61.7%
BVD status (% of herds)	Free	45.4%
	Non-free or unknown	54.6%
Paratuberculosis status (% of herds)	Unsuspected	80.5%
	Suspected	19.5%
Salmonella status (% of herds)	Unsuspected	93.1%
	Suspected	6.9%
Milk price/kg	Continuous	€0.34
Slaughter cow price/kg	Continuous	€2.82
Replacement cow price^#^	Continuous	€928
Annual cattle replacement rate (mean % per herd)	10% lowest replacement	14.9%
	40% lower replacement	22.3%
	40% higher replacement	29.7%
	10% highest replacement	41.5%
Purchase of cattle in the previous year (% of herds)	Yes, >2 cattle/year	38.2%
	Yes, 1–2 cattle/year	10.5%
	No	51.2%
Season	Winter (Jan-Mar)	27.2%
	Spring (Apr-Jun)	23.8%
	Summer (Jul-Sep)	27.4%
	Autumn (Oct-Dec)	21.6%
Milk production level at herd level (mean yearly net revenue^+^; € per cow)	10% lowest	€1.577
	40% lower	€2.058
	40% higher	€2.393
	10% highest	€2.706
Herd size (mean number of cattle >2 years of age)	10% smallest herds	33.0
	40% smaller herds	69.9
	10% larger herds	122.5
	10% largest herds	244.8
Location of herd (province^*^) (% of herds)	Drenthe (North)	3.8%
	Flevoland (North)	2.1%
	Friesland (North)	7.0%
	Groningen (North)	3.2%
	Overijssel (East)	10.7%
	Gelderland (East)	15.7%
	N-Holland (West)	4.6%
	Utrecht (West)	8.3%
	Z-Holland (West)	10.0%
	Limburg (South)	5.4%
	N-Brabant (South)	26.5%
	Zeeland (South)	2.7%

# *Dairy cows, 1st class, producer price per animal*.

+ *Standardized milk production (Dutch Royal Cattle Syndicate (CRV), Arnhem, the Netherlands)*.

* *The region in the Netherlands where the province is located is mentioned for clarity*.

Associations between frequencies of AM- or PM-findings and independent variables were expressed as exponentiated coefficients (i.e., incidence rate ratios; IRR). For example: an explanatory variable with an IRR of 1.11 means that a unit increase in the explanatory variable corresponds to an increase of 11% in the number of cattle with a finding of category × per herd per quarter. Statistical significant IRR's ≤0.8 or ≥1.25 were deemed epidemiologically relevant.

## Results

### Representativeness

**Figure 1:**
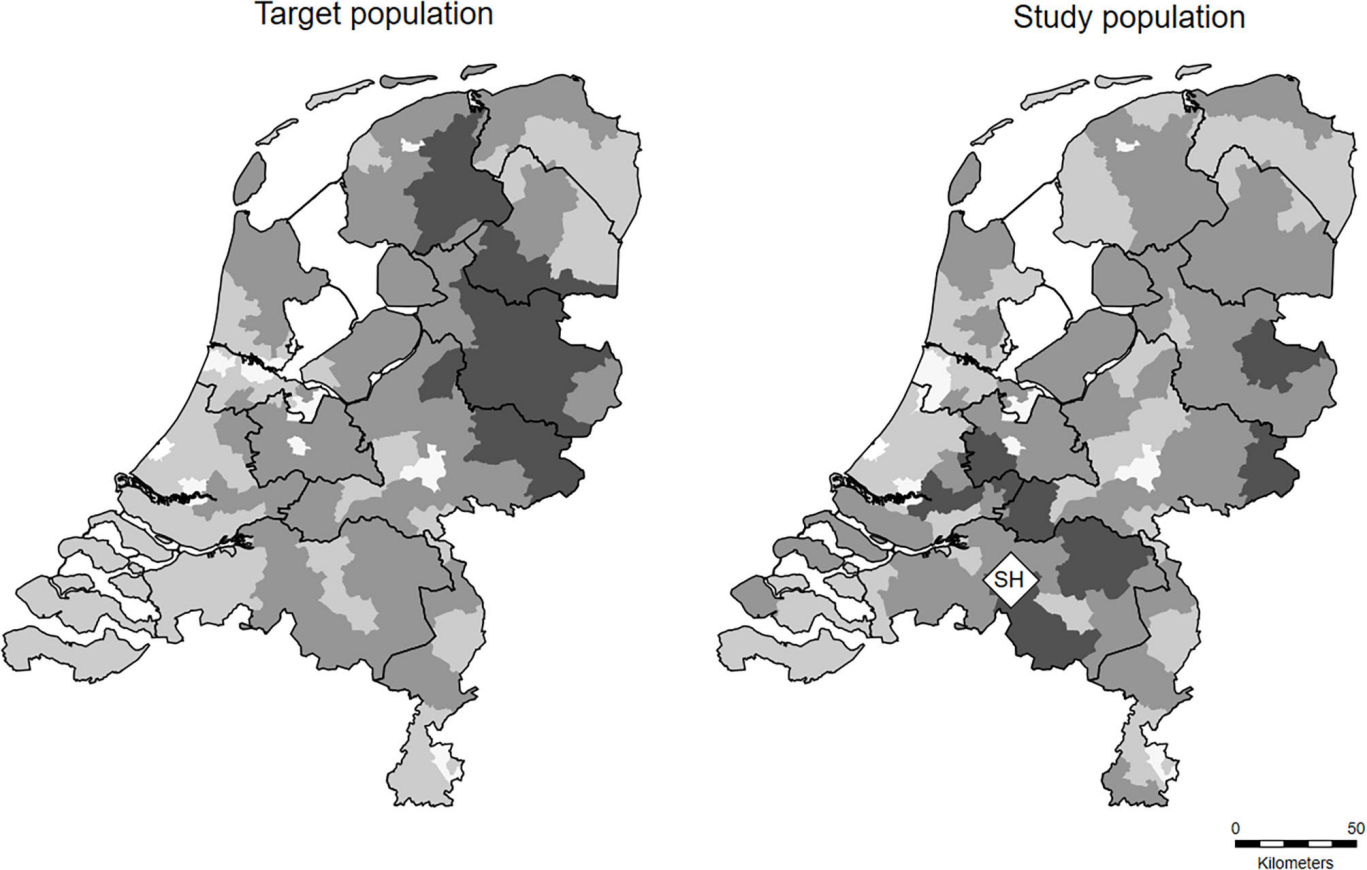
Regional distribution of dairy herds in the target population and the study population per two-digit postal district over the period 2015–2018, with the slaughterhouse of study indicated with a diamond symbol (“SH”). Increasing color intensity of areas correspond with increasing cattle herd density.

About 90% of the meat inspection records originated from dairy herds (*N* = 10,406 herds, *n* = 422,194 slaughtered cattle). Inspection of the characteristics of these herds in relation to the overall dairy cattle population lead to the conclusion that the dairy herds in the slaughterhouse data were sufficiently representative for the Dutch dairy population (Table 2). However, the regional distribution of the dairy herds in the slaughterhouse data was skewed toward the southern region, probably due to the location of the slaughterhouse in the south of the Netherlands (Figure 1).

**Table 2:** Characteristics of dairy herds in the slaughterhouse dataset (study population) and all dairy herds in the Netherlands (target population) between January 2015 and September 2018.

	**Herd size (mean)**			**Farming system**	**Herd health status**	
	**0–1 year**	**1–2 year**	**>2 year**	**Closed**	**BVD-free**	**BHV-1-free**
Target population (*N* = 17,263)	36	29	103	52%	43%	38%
Study population (*N* = 10,406)	41	32	113	51%	45%	38%

### Descriptive Results

AM-findings were less common than PM-findings (Table 3). About 92% of the slaughtered cattle had no AM-finding and 45% of the slaughtered cattle had no PM-finding. AM-categories that were not further analyzed due to their very low frequency were findings related to the lung/heart, body condition, locomotion, skin/mucosa, udder, birth canal, digestion, and welfare. PM-findings related to liver, pulmonary/peritoneal membrane, and integumentary lesions were most common amongst the PM-categories. PM-findings related to the back and neck were omitted from further analyses due to their low frequency of occurrence amongst PM-categories.

**Table 3:** Mean yearly percentage of slaughtered cattle with an AM- or PM-finding per category in one large Dutch slaughterhouse between January 1, 2015 and September 30, 2018. Categories that were not further analyzed are displayed in gray. N = 125.006 slaughtered cattle per year.

**AM-category**	**%**	**PM-category**	**%**
Body condition	0.99	Condemnation	2.46
Lung/heart	0.20	Liver fluke	6.41
Locomotion	0.59	Liver (except liver fluke)	11.86
Skin/mucosa	1.82	Lungs	7.82
Hygiene	4.01	Pulmonary membrane/peritoneum	14.20
Udder	0.08	heart	3.03
Birth canal	0.00	Udder	9.12
Digestion	0.30	Kidneys	4.82
Welfare	0.01	Lesions of the integumentary system	10.15
		Back	0.57
		Neck	2.06
		Round/buttock region	6.62
		Gastrointestinal tract	2.40
No AM-findings	92.48	No PM-findings	45.41

### Multivariable Results

#### Associations With AM-Findings and Trend in Time

**Figure 2:**
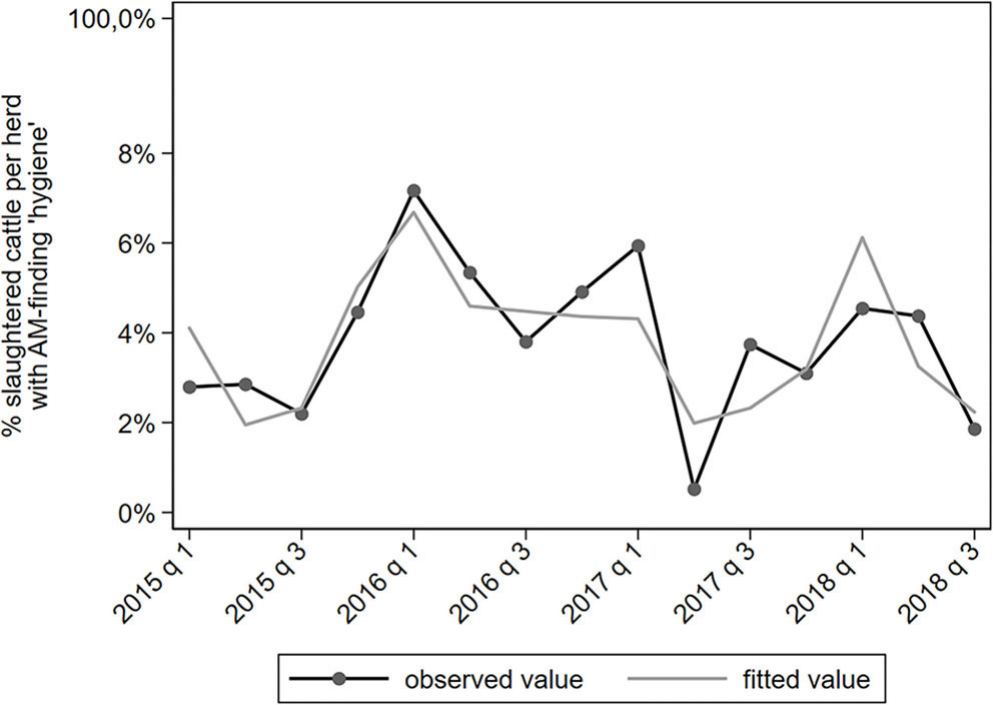
Observed and predicted percentage of slaughtered cattle per dairy herd with a AM-finding categorized under “hygiene,” per quarter between January 2015 to September 2018 (*N* = 78,366).

**Figure 3:**
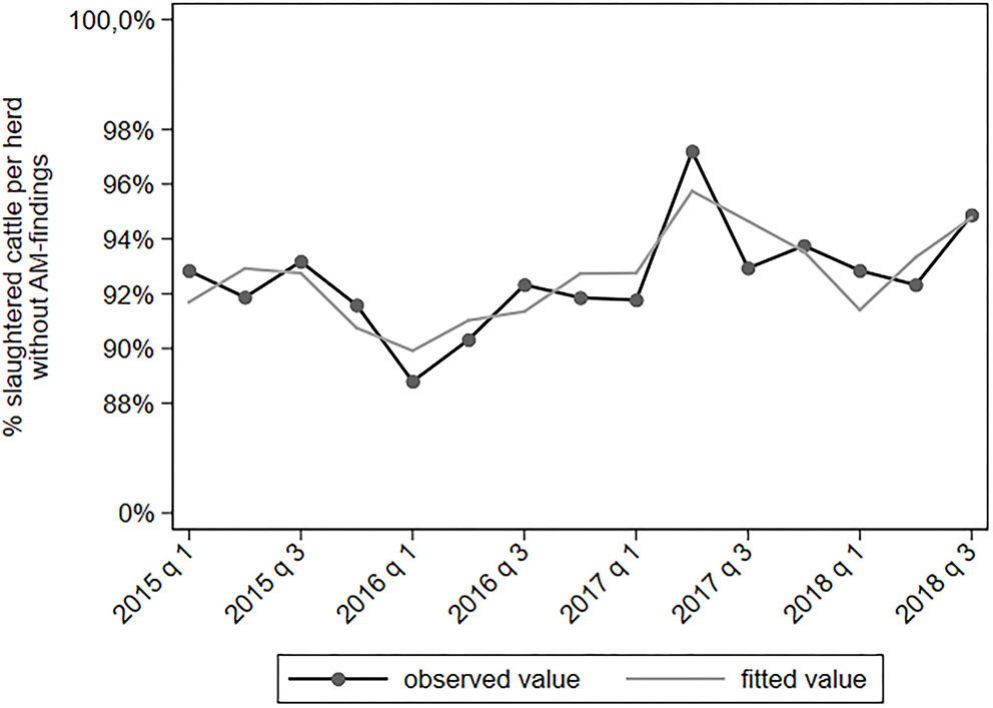
Observed and predicted percentage of slaughtered cattle per dairy herd without AM-findings, per quarter between January 2015 to September 2018 (*N* = 78,366).

Results of the multivariable analyses of AM-findings are summarized in Table 4. Only statistical significant associations with a IRR ≤0.8 or ≥1.25 are shown. A complete overview of all associations is provided in Supplementary Table 1. The proportion of slaughtered cattle with an AM-finding related to hygiene varies a lot in time, which could not be captured well by the model (Figure 2). Due to this suboptimal fit of the model, the results of the AM-finding “hygiene” should be interpreted with caution. The proportion of slaughtered cattle in which no AM-findings were found shows an increasing trend in time (Figure 3). There were no epidemiologically relevant associations with explanatory variables (Table 4).

**Table 4:** Results of multivariable analyses of AM-findings amongst slaughtered cattle from dairy herds between January 1, 2015 and September 30, 2018, in incidence ratios (IRR) and 95% confidence intervals. Only associations with a p-value < 0.05 that met the relevance criteria (IRR ≤0.8 or ≥1.25) are shown (N = 78,366).

	**AM-category**	**Hygiene**	**No AM-finding**
**Explanatory variable**		**IRR**	**IRR**
Slaughter cow price (€/kg)		3.57 (2.81–4.54)	–
Season			
Mean	Ref.	Ref.	
	Winter (Jan-Mar)	1.51 (1.47–1.56)	–
	Spring (Apr-Jun)	0.52 (0.50–0.55)	–
	Summer (Jul-Sep)	0.70 (0.68–0.73)	–
	Autumn (Oct-Dec)	1.80 (1.72–1.90)	–

#### Associations With PM-Findings and Trend in Time

Results of the multivariable analyses of PM-findings are summarized in Table 5. Only statistical significant associations with a IRR ≤0.8 or ≥1.25 are shown. A complete overview of all associations is provided in Supplementary Table 2. Due to the large number of meat inspection categories analyzed, only a selection of the trends in time and relevant associations are described below.

**Table 5:** Results of multivariable analyses of PM-findings amongst slaughtered cattle from dairy herds between January 1, 2015 and September 30, 2018, in incidence ratios (IRR) and 95% confidence intervals. Only associations with a p-value < 0.05 that met the relevance criteria (IRR ≤0.8 or ≥1.25) are shown (N = 78,366).

**PM-category**	**Condemnation**	**Lungs**	**Pulmonary membrane/ peritoneum**	**Heart**	**Liver fluke**	**Liver (ex. Liver fluke)**	**Kidneys**	**Udder**	**Integumentary**	**Round/ buttock**	**Gastrointestinal tract**	**No PM-findings**
**Explanatory variable**	**IRR**	**IRR**	**IRR**	**IRR**	**IRR**	**IRR**	**IRR**	**IRR**	**IRR**	**IRR**	**IRR**	**IRR**
Slaughter cow price (€/kg)	–	0.78 (0.69–0.88)	1.38 (1.25–1.52)	0.73 (0.60–0.89)	1.63 (1.39–1.90)	–	–	3.17 (2.79–3.60)	–	–	1.32 (1.04–1.68)	–
Milk production level at herd level (mean yearly net revenue; € per cow)												
Mean	Ref.	Ref.	Ref.	Ref.	Ref.	Ref.	Ref.	Ref.	Ref.	Ref.	Ref.	Ref.
10% lowest	–	–	–	–	1.27 (1.19–1.35)	–	–	–	–	–	–	–
40% lower	–	–	–	–	–	–	–	–	–	–	–	–
40% higher	–	–	–	–	–	–	–	–	–	–	–	–
10% highest	–	–	–	–	–	–	–	–	–	–	–	–
Missing	–	–	–	–	–	–	–	–	–	–	–	–
Herd size (mean number of cattle >2 years of age)												
Mean	Ref.	Ref.	Ref.	Ref.	Ref.	Ref.	Ref.	Ref.	Ref.	Ref.	Ref.	Ref.
10% smallest herds	0.74 (0.67–0.83)	–	0.80 (0.76–0.83)	–	–	–	–	–	–	–	–	–
40% smaller herds	–	–	–	–	–	–	–	–	–	–	–	–
10% larger herds	–	–	–	–	–	–	–	–	–	–	–	–
10% largest herds	1.26 (1.19–1.34)	–	–	–	–	–	–	–	–	–	–	–
Location of herd (province)												
Mean	Ref.	Ref.	Ref.	Ref.	Ref.	Ref.	Ref.	Ref.	Ref.	Ref.	Ref.	Ref.
Drenthe	0.79 (0.68–0.92)	–	–	–	0.70 (0.64–0.77)	–	–	–	–	–	–	–
Flevoland	–	–	–	–	–	–	–	–	–	–	–	-
Friesland	0.47 (0.40–0.54)	–	–	–	–	–	–	–	0.78 (0.74–0.83)	–	–	–
Gelderland	–	–	–	–	–	–	–	–	–	–	–	–
Groningen	0.66 (0.56–0.78)	–	–	–	0.72 (0.61–0.85)	–	–	–	–	–	–	–
Limburg	–	–	–	–	–	–	–	–	–	–	–	–
N-Brabant	1.38 (1.31–1.46)	–	–	–	0.67 (0.64–0.70)	–	–	–	1.27 (1.24–1.31)	–	–	–
N-Holland	1.26 (1.14–1.39)	–	–	–	1.97 (1.84–2.12)	–	–	–	–	–	–	–
Overijssel	–	–	–	–	0.73 (0.68–0.78)	–	–	–	–	–	–	–
Utrecht	1.50 (1.38–1.64)	–	–	–	1.70 (1.61–1.80)	–	–	–	–	–	–	–
Z-Holland	1.37 (1.26–1.48)	–	–	–	2.27 (2.16–2.38)	–	–	–	–	–	–	–
Zeeland	–	–	–	–	–	–	–	–	–	–	–	–

**Figure 4:**
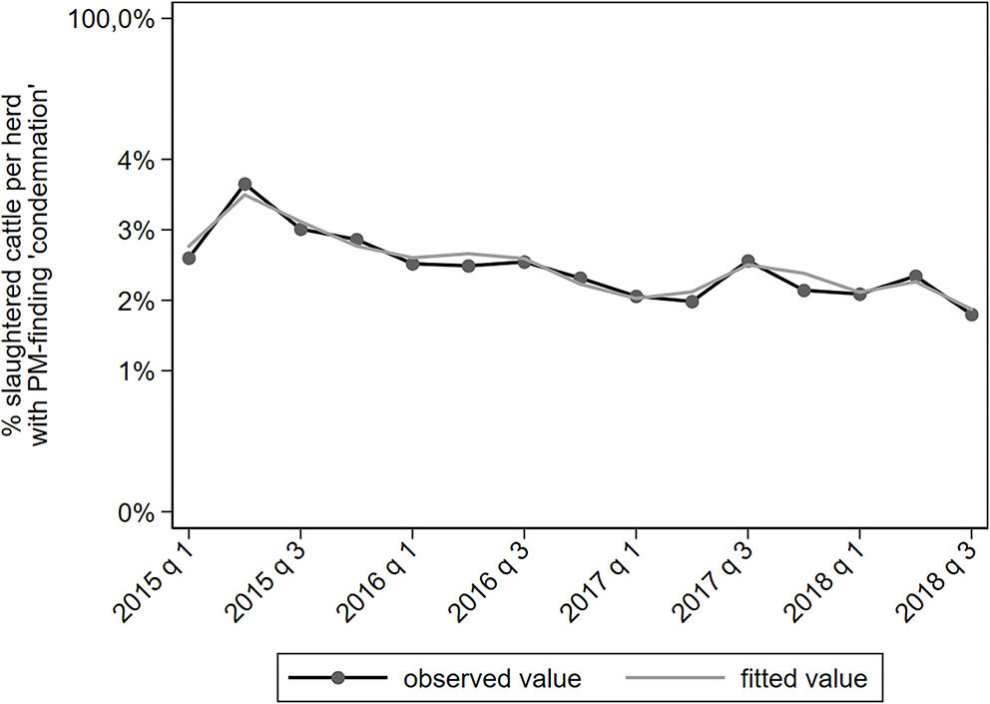
Observed and predicted percentage of slaughtered cattle per dairy herd with a PM-finding categorized under “condemnation,” per quarter between January 2015 to September 2018 (*N* = 78,366).

The proportion of cattle with the PM-finding “condemnation” gradually decreased in time (Figure 4). Cattle from small herds had a lower proportion of carcass condemnation (IRR = 0.74 for the 10% smallest herds) and cattle from large herds had a higher proportion of carcass condemnation (IRR = 1.26 for the 10% largest herds). To illustrate this: the 10% largest herds had 26% more cattle sent to slaughter with carcass condemnation than the average herd in the dataset. Herds from the northern provinces Drenthe, Friesland and Groningen had a lower proportion carcass condemnations than the average farm (IRR = 0.46–0.79). Herds from central and southern provinces had a higher proportion of carcass condemnations (IRR = 1.26–1.50).

**Figure 5:**
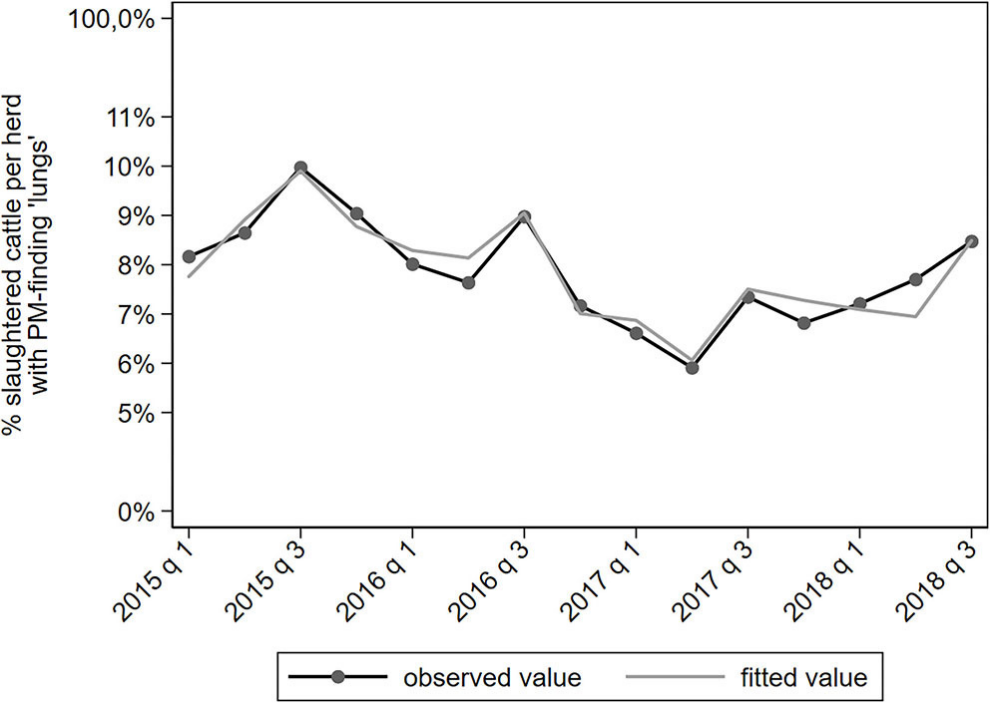
Observed and predicted percentage of slaughtered cattle per dairy herd with a PM-finding categorized under “lungs,” per quarter between January 2015 to September 2018 (*N* = 78,366).

PM-findings categorized under “lungs” mainly represent pneumonia. An increase in slaughter cow price was associated with a lower proportion of cattle with a PM-finding related to lungs (IRR = 0.78). The proportion of cattle with a PM-finding related to lungs was decreasing until mid-2017 but has been increasing since (Figure 5).

**Figure 6:**
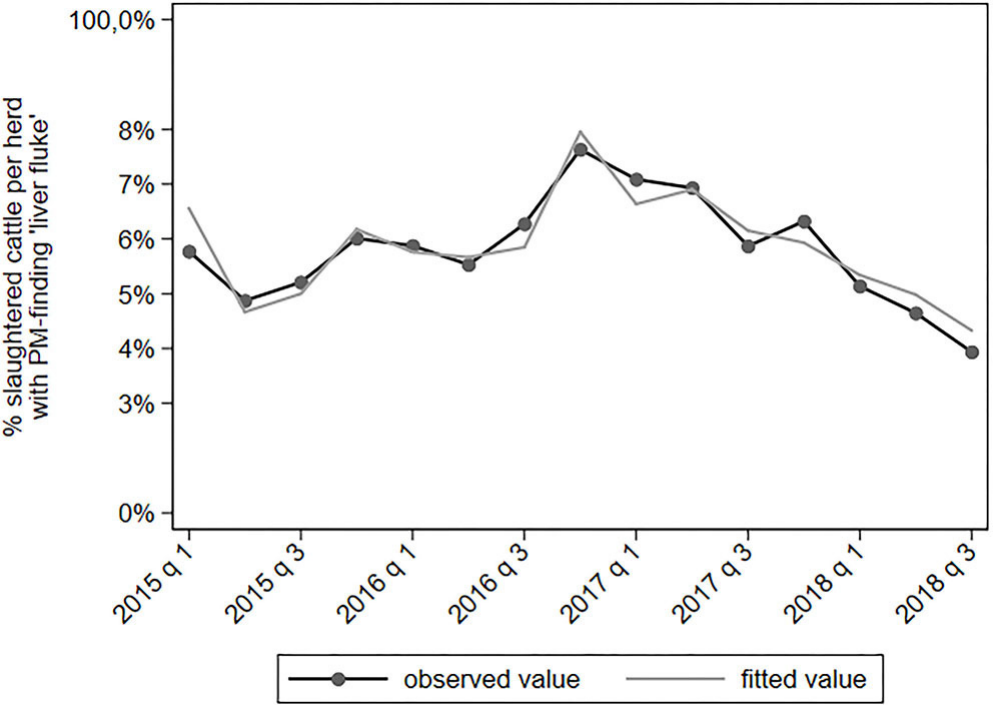
Observed and predicted percentage of slaughtered cattle per dairy herd with a PM-finding categorized under “liver fluke,” per quarter between January 2015 to September 2018 (*N* = 78,366).

The proportion of cattle with a PM-finding “liver fluke” decreased since 2018 (Figure 6). Post-mortem liver fluke findings are the result of both acute and past infections and a distinction could not be made from the data. Cattle from herds with a low milk production had a higher proportion of slaughtered cattle with a liver fluke finding (IRR = 1.27 for the 10% least producing herds). Herds located in central and western provinces had an increased risk of PM-finding “liver fluke” (IRR = 1.70–2.27).

**Figure 7:**
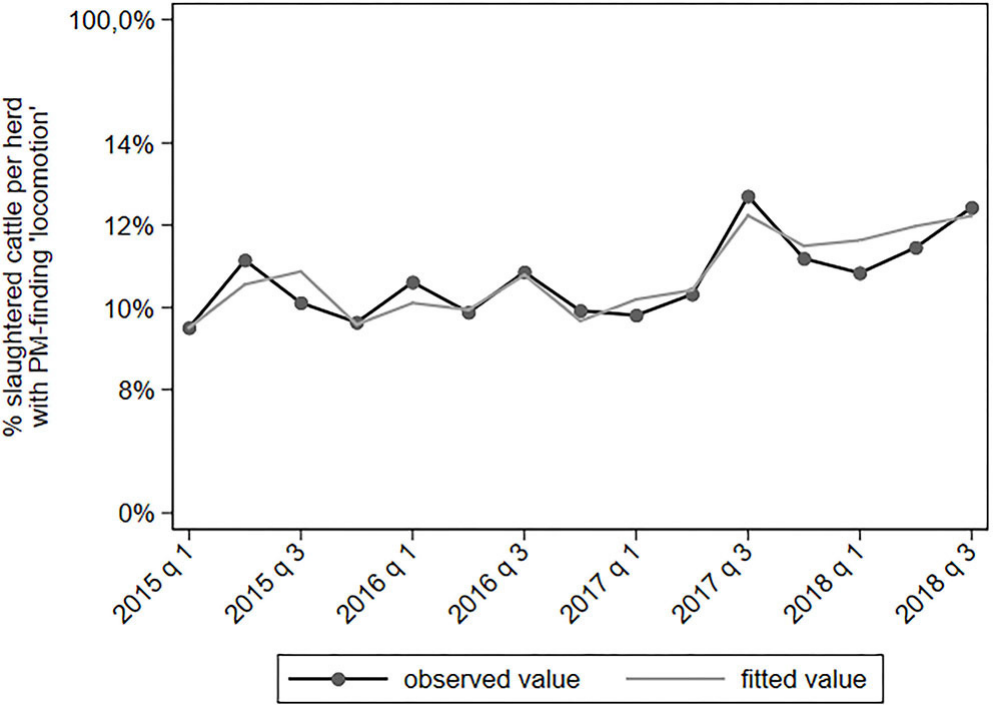
Observed and predicted percentage of slaughtered cattle per dairy herd with a PM-finding categorized under “integumentary,” per quarter between January 2015 to September 2018 (*N* = 78,366).

PM-findings classified as “lesions of the integumentary system” include lesions in the hock, hip, knee, shoulder, or front leg. The proportion of cattle with a PM-finding related to integumentary lesions increased in time since 2017q3 (Figure 7). Herds from the north of the Netherlands had a lower proportion of such findings than the average farm (IRR = 0.77). Herds from southern provinces had a higher proportion of such findings (IRR = 1.31–1.36).

**Figure 8:**
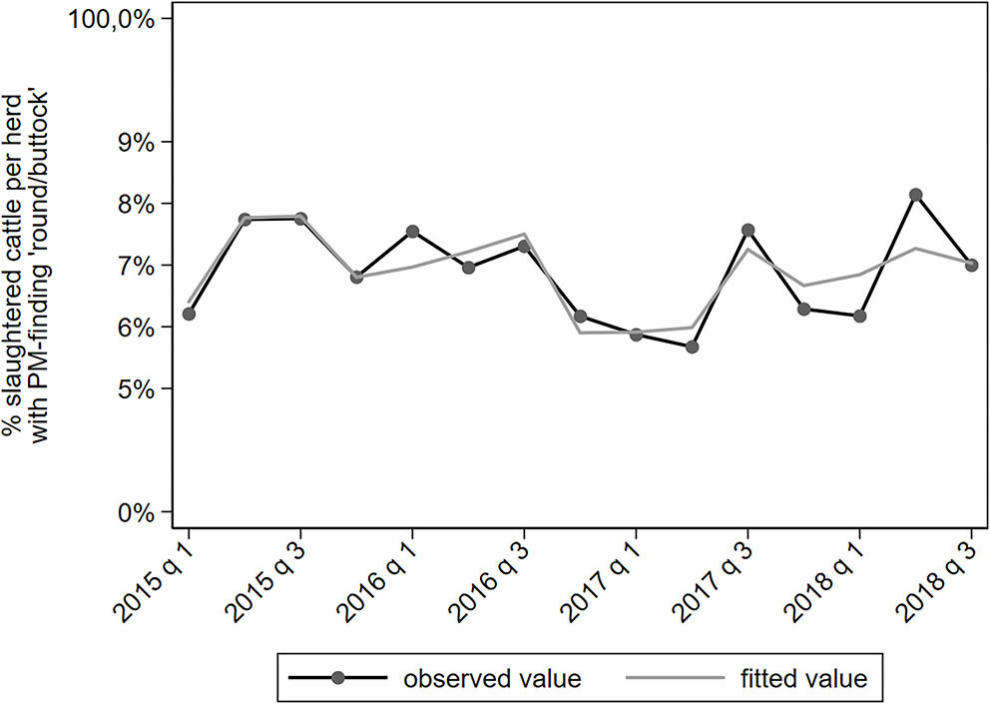
Observed and predicted percentage of slaughtered cattle per dairy herd with a PM-finding categorized under “round/buttock region,” per quarter between January 2015 to September 2018 (*N* = 78,366).

PM-findings categorized as “round and buttock region” represent internal trauma and injuries in that part of the carcass. These can be caused in the herd of origin or during transport. The proportion of slaughtered cattle with such PM-findings fluctuated around 7% per herd per quarter (Figure 8). There were significant but no relevant associations with explanatory variables.

## Discussion

Meat inspection records of one large cattle slaughterhouse were analyzed in this study to evaluate the added value of slaughterhouse data for cattle health surveillance in the Netherlands. “Added value” was defined as health indicators that provide information regarding specific health problems, or new information not yet available in the TASC.

### Representativeness of the Study Population

The characteristics of the dairy herds in the slaughterhouse data did not differ from the overall dairy cattle population. Therefore, the dairy herds in the slaughterhouse data were considered sufficiently representative for the Dutch dairy population. However, the distribution of the dairy herds in the slaughterhouse data was skewed toward the southern region, probably due to the location of the slaughterhouse in the south of the Netherlands.

### Associations With Risk Factors

The model results revealed a number of noteworthy associations between farm of origin characteristics and the occurrence of meat inspection findings (“risk factors”). The objective of the TASC is to monitor trends and developments in cattle health, rather than case detection, yet the risk factors that were found may illustrate a profile of high-risk herds. This could lead to a more risk-based surveillance approach.

Carcasses of cows that originated from the 10% smallest dairy herds were less likely to be condemned than the sample mean, and carcasses that that originated from the 10% largest herds were more likely to be condemned. This is in agreement with a study by (5) on risk factors for whole carcass condemnation of slaughtered cattle in Switzerland. Also carcasses from the 10% smallest herds had less often a PM-finding related to the pulmonary membrane/peritoneum. One explanation for this could be that some of these findings are the result of infectious diseases which are less likely in smaller herds (8, 9).

PM-findings categorized under “liver fluke” were observed more often in the provinces N-Holland, Z-Holland, and Utrecht. These provinces are known as high-risk liver fluke areas in the Netherlands (10). Condemnation and PM-findings categorized under “integumentary” were observed less often in carcasses from cows that originated from the (northern) provinces with the farthest distance to the slaughterhouse (located in the south). This is somewhat surprising as it is suggested that injuries such as bruising might increase with the distance traveled by cattle (11). It is possible however that healthy cattle are transported over a longer distance than cattle that are less healthy, i.e., those are expected to be transported to a nearby slaughterhouse. Yet as a consequence, the association between farm of origin location and occurrence of certain PM-findings (“integumentary” and “condemnation”) were probably biased to some extent by the location of the slaughterhouse and should be interpreted with caution. Inclusion of meat inspection data from slaughterhouses in other regions is therefore of importance to assure sufficient regional coverage.

Slaughter prices vary constantly and are associated with live cattle prices and milk prices. For example, farmers are more driven to send cows to slaughter when milk prices are low, creating a greater supply of slaughter cows and consequently a decrease in slaughter cow price. This process is enhanced by changes in agricultural policy. During the study period, two major policy changes occurred in the Netherlands: (1) the abolishment of milk-quota in 2015 and (2) the introduction of the Phosphate Regulation in 2017. These events have undoubtedly influenced farmer's culling decisions and possibly the health status of the slaughtered cattle population. In our study, slaughter cow price was included in the model as the mean national slaughter cow price per quarter of the year. The association between slaughter cow price and the probability of AM- and PM-findings was ambiguous, varying from an IRR of 0.73 to 3.57. This suggests that the quarterly slaughter cow prices did not capture the true relation between fluctuations in supply and demand of slaughter cows and their health status (expressed as the presence of AM- or PM-findings).

Finally, the model we used could not adequately describe the trend of some meat inspection findings, such as the AM-finding “hygiene.” One reason for this could be that important explanatory variables are missing, such as type of farming system (conventional/organic) and whether or not grazing is applied. Unless this lack of fit is resolved, inadequately described meat inspection findings are unsuitable to be added to the TASC.

### Trends of Meat Inspection Findings in Time

In this study, meat inspection records were translated into trends of the proportion of slaughtered cattle with AM- and/or PM-findings, resulting in meaningful indicators of cattle health. From the 16 categories analyzed, seven were deemed of added value to analyses in the existing trend analysis surveillance component. First, the PM-category “condemnation” is of relevance due to the severe character of this finding, although, there is a high diversity of possible reasons for carcass condemnation (12). The PM-categories “integumentary” and “round/buttock region” are relevant for cattle health surveillance as a welfare measure, which is not yet covered by the TASC. Thus, these results provide new information as such. The PM-categories “lungs” and “liver fluke” provide information regarding specific health problems such as respiratory disorders and fasciolosis. The proportion of cattle with a PM-finding “liver fluke” decreased since 2018 which is in agreement with a decrease in active liver fluke infections in dairy herds as a result of the dry summer of 2018 (unpublished data). The increasing trend in PM-findings categorized as “lungs” was unexpected however and is an example of an abnormal change that could be a reason for more in-depth investigation. Also, these results serve complementary to signals derived from other surveillance activities such as necropsy examinations of fallen stock. Finally, the categories “no AM-findings” and “no PM-findings” could serve as a potential favorable measure of animal health.

If meat inspection data were to be added to the existing TASC, results will be reported to a national steering committee on a quarterly basis, together with other indicators of cattle health that are part of the TASC (1). Possible causes of deviating trends may be investigated in more detail on request of the steering committee. An example of this process is the initiative to investigate reasons for the increased calf mortality in the Dutch dairy sector that was observed in 2009–2010, after a period of several years in which calf mortality rates remained stable (13). Another application of meat inspection data could be in the form of real-time spatiotemporal analyses, providing an opportunity for early-warning (syndromic) surveillance systems. This could be particularly interesting for diseases for which post-mortem lesions are more specific than clinical symptoms (12).

### Challenges for Implementation

Slaughterhouse data could be a valuable source of information of herd types of which cattle health information is scarce, such as small-scale holders. Unfortunately, non-dairy herds were underrepresented in the dataset that was used for this study. This underlines the need for data from other slaughterhouses before implementation of meat inspection data analyses in the current surveillance system. However, the lack of standardization between slaughterhouses in recording of inspection findings presents challenges for implementation (14). In addition, although, official veterinarians and their auxiliaries are trained according to a standardized inspection protocol, the meat inspection remains a subjective judgement to some extent. Also, factors such as experience, motivation and dedication as well as local operational aspects impact the compliance with inspection protocols (15). As a result, diagnostic performance and inter-inspector variability are known challenges of meat inspection (15, 16) and bias apparent prevalences of meat inspection findings (17). It is expected however that this bias is rather constant over time, thus meaningful trends may still be derived from meat inspection data. Nevertheless, these issues need to be taken into account when using slaughterhouse data for cattle health surveillance.

## Conclusion

Categorizing and analyzing routinely collected meat inspection data as herd-level frequencies of ante-mortem and post-mortem findings yields valuable cattle health indicators at population level. A number of indicators yields information that is not captured in other Dutch census data sources used in the national surveillance programme, or provides improved understanding when combined with signals from other surveillance components. Based on this study, stakeholders were advised to explore the availability of data from other slaughterhouses to improve the regional coverage and representation of various herd types to enable implementation of meat inspection data analyses in the cattle health surveillance system in the Netherlands.

## Data Availability Statement

The data analyzed in this study is subject to the following licenses/restrictions: The datasets generated for this study will not be made publicly available as the data has been provided by a commercial abattoir, who wishes not to make their data publicly available. Requests to access these datasets should be directed to a.veldhuis@gdanimalhealth.com.

## Author Contributions

AV and GS developed the statistical models. AV performed the statistical analyses and wrote the first draft of the manuscript. All authors contributed to the conception and design of the study, interpretation of the results, manuscript revision, read, and approved the submitted version.

## Conflict of Interest

AV, DS, HW, and GS are employed by company Royal GD. MB is employed by company Vion.
