# Supplementary material for: Added Value of Meat Inspection Data for Monitoring of Dairy Cattle Health in the Netherlands
Source: Front Vet Sci. 2021 Jul 15;8:661459. doi: 10.3389/fvets.2021.661459 (PMC8319994; doi:10.3389/fvets.2021.661459)
Supplement: Supplementary file 1 [file Table_1.DOCX]

**Supplementary Table 1.** Results of multivariable analyses of AM-findings amongst slaughtered cattle from dairy herds between January 1, 2015 and September 30, 2018. Associations with a *p-*value <0.05 are displayed in bold.

| *AM-category* | *hygiene* | *no AM-finding* |
| --- | --- | --- |
| *Explanatory variable* | IRR | IRR |
| Quarter-year | **1.07** | **1.00** |
| Age at slaughter | **1.00** | **1.00** |
| Antibiotic use in cattle 1-2 years of age No | Ref. | Ref. |
| Yes | 0.99 | 1.00 |
| Antibiotic use in cattle >2 years of age Mean | Ref. | Ref. |
| 10% herds with lowest DDDA | **0.88** | **1.01** |
| 40% herds with lower DDDA | 1.03 | 1.00 |
| 40% herds with higher DDDA | **1.06** | **1.00** |
| 10% herds with highest DDDA | 1.05 | **1.00** |
| BHV-1 status  Non-free or unknown  Free | Ref.  0.96 | Ref.  **1.01** |
| BVD-status  Non-free or unknown  Free | Ref.  0.99 | Ref.  1.00 |
| Salmonella status  Unsuspected  Suspected | Ref.  1.04 | Ref.  **0.99** |
| Paratuberculosis-status  Suspected  Unsuspected | Ref.  1.00 | Ref.  **1.00** |
| Purchase of cattle in the previous year Yes, >2 cattle/year | Ref. | Ref. |
| Yes, 1-2 cattle/year | 1.03 | 1.00 |
| No | 1.00 | 1.00 |
| Milk price (€ / kg) | **0.89** | **1.00** |
| Slaughter cow price (€ / kg) | **3.57** | **1.02** |
| Replacement cow price (€) | **1.00** | 1.00 |
| Growth in herd size Mean | Ref. | Ref. |
| 10% least growth | **0.92** | **1.01** |
| 40% less growth | 1.01 | 1.00 |
| 40% more growth | **1.04** | **1.00** |
| 10% most growth | 1.04 | **1.00** |
| Annual replacement rate Mean | Ref. | Ref. |
| 10% least replacement | **0.93** | **1.01** |
| 40% less replacement | 1.00 | 1.00 |
| 40% more replacement | 1.01 | **1.00** |
| 10% most replacement | **1.06** | **0.99** |
| Season Mean | Ref. | Ref. |
| Winter (Jan-Mar) | **1.51** | **0.99** |
| Spring (Apr-Jun) | **0.52** | **1.01** |
| Summer (Jul-Sep) | **0.70** | **1.01** |
| Autumn (Oct-Dec) | **1.80** | **0.99** |
| Milk production level at herd level (mean yearly net revenue; € per cow) Mean | Ref. | Ref. |
| 10% lowest | **1.13** | **0.99** |
| 40% lower | **1.04** | **1.00** |
| 40% higher | **0.92** | **1.00** |
| 10% highest | **0.87** | **1.01** |
| missing | 1.05 | 1.00 |
| Herd size (mean number of cattle >2 years of age) Mean | Ref. | Ref. |
| 10% smallest herds | 1.04 | **1.01** |
| 40% smaller herds | 1.01 | 1.00 |
| 10% larger herds | 0.98 | 1.00 |
| 10% largest herds | 0.97 | **1.00** |
| Location of herd (province) Mean | Ref. | Ref. |
| Drenthe | 1.04 | 1.00 |
| Flevoland | **0.84** | **1.01** |
| Friesland | **0.88** | **1.02** |
| Gelderland | 1.01 | 1.00 |
| Groningen | 1.07 | **1.01** |
| Limburg | 1.06 | **0.99** |
| N-Brabant | **1.17** | **0.98** |
| N-Holland | 0.96 | 1.00 |
| Overijssel | **0.83** | **1.01** |
| Utrecht | **1.08** | **0.99** |
| Z-Holland | **1.19** | **0.99** |
| Zeeland | 0.95 | 1.00 |
