# Supplementary material for: Added Value of Meat Inspection Data for Monitoring of Dairy Cattle Health in the Netherlands
Source: Front Vet Sci. 2021 Jul 15;8:661459. doi: 10.3389/fvets.2021.661459 (PMC8319994; doi:10.3389/fvets.2021.661459)
Supplement: Supplementary file 2 [file Table_2.DOCX]

**Supplementary Table 2.** Results of multivariable analyses of PM-findings amongst slaughtered cattle from dairy herds between January 1, 2015 and September 30, 2018. Associations with a p-value <0.05 are displayed in bold.

| *PM-category* | *condemnation* | *lungs* | *pulmonary membrane/peritoneum* | *hart* | *liver fluke* | *liver (ex. liver fluke)* | *kidneys* | *udder* | *integumentary* | *round /buttock* | *gastrointestinal tract* | *no PM-findings* |
| --- | --- | --- | --- | --- | --- | --- | --- | --- | --- | --- | --- | --- |
| *Explanatory variable* | IRR | IRR | IRR | IRR | IRR | IRR | IRR | IRR | IRR | IRR | IRR | IRR |
| Quarter-year | **0.97** | **0.98** | 1.00 | **0.97** | **0.99** | **0.98** | 0.99 | **0.99** | **1.02** | **1.00** | **0.98** | 1.01 |
| Age at slaughter | **1.01** | **1.00** | **1.01** | **1.00** | **1.00** | **1.00** | **1.02** | **1.00** | **1.01** | **1.01** | **1.00** | **0.99** |
| Antibiotic use in cattle 1-2 years of age No | Ref. | Ref. | Ref. | Ref. | Ref. | Ref. | Ref. | Ref. | Ref. | Ref. | Ref. | Ref. |
| Yes | **1.08** | 1.00 | **1.04** | 1.01 | **1.09** | 0.99 | 1.01 | 1.00 | 1.00 | 1.00 | 0.99 | **0.98** |
| Antibiotic use in cattle >2 years of age Mean | Ref. | Ref. | Ref. | Ref. | Ref. | Ref. | Ref. | Ref. | Ref. | Ref. | Ref. | Ref. |
| 10% herds with lowest DDDA | **0.85** | **0.95** | 1.01 | **0.91** | **1.11** | **0.96** | 0.93 | 1.01 | **0.92** | **0.92** | 0.98 | **1.02** |
| 40% herds with lower DDDA | 1.00 | 0.99 | 0.99 | 1.02 | 0.98 | 1.01 | **0.95** | 0.99 | 1.00 | 0.99 | 0.99 | **1.01** |
| 40% herds with higher DDDA | **1.07** | 1.02 | 1.00 | **1.04** | **0.97** | 1.00 | 1.02 | 1.00 | 1.02 | 1.00 | 1.01 | 0.99 |
| 10% herds with highest DDDA | **1.09** | **1.04** | 1.00 | 1.04 | **0.95** | **1.03** | **1.10** | 1.00 | **1.07** | **1.10** | 1.03 | **0.98** |
| BHV-1 status  Non-free or unknown  Free | Ref.  **0.88** | Ref.  0.98 | Ref.  **0.95** | Ref.  **0.96** | Ref.  1.02 | Ref.  0.99 | Ref.  **0.93** | Ref.  1.00 | Ref.  **0.96** | Ref.  **0.91** | Ref.  **0.95** | Ref.  **1.03** |
| BVD-status  Non-free or unknown  Free | Ref.  0.97 | Ref.  0.98 | Ref.  **0.97** | Ref.  0.99 | Ref.  0.98 | Ref.  0.99 | Ref.  0.98 | Ref.  0.99 | Ref.  **0.97** | Ref.  1.00 | Ref.  1.02 | Ref.  **1.01** |
| Salmonella status  Unsuspected  Suspected | Ref.  **1.12** | Ref.  0.98 | Ref.  **1.07** | Ref.  **0.91** | Ref.  **1.14** | Ref.  0.99 | Ref.  **1.07** | Ref.  1.01 | Ref.  1.01 | Ref.  1.01 | Ref.  1.01 | Ref.  **0.97** |
| Paratuberculosis-status  Suspected  Unsuspected | Ref.  **0.91** | Ref.  0.98 | Ref.  **0.94** | Ref.  1.00 | Ref.  0.97 | Ref.  **0.98** | Ref.  **0.93** | Ref.  0.98 | Ref.  1.01 | Ref.  1.03 | Ref.  0.97 | Ref.  **1.02** |
| Purchase of cattle in the previous year Yes, >2 cattle/year | Ref. | Ref. | Ref. | Ref. | Ref. | Ref. | Ref. | Ref. | Ref. | Ref. | Ref. | Ref. |
| Yes, 1-2 cattle/year | **0.88** | 1.00 | **0.89** | 1.01 | 0.97 | 0.99 | **0.94** | 0.98 | 0.99 | **0.96** | 0.96 | **1.03** |
| No | 0.94 | 0.99 | **0.97** | 0.97 | **1.09** | 1.00 | 0.98 | 1.00 | 0.99 | 0.97 | 1.00 | 1.01 |
| Milk price (€ / kg) | 0.99 | **0.98** | 1.00 | **0.99** | **1.03** | 1.00 | **0.99** | **0.95** | 1.00 | **0.97** | 1.00 | **1.00** |
| Slaughter cow price (€ / kg) | **1.20** | **0.78** | **1.38** | **0.73** | **1.63** | 0.93 | 0.87 | **3.17** | 1.02 | 1.10 | **1.32** | **0.81** |
| Replacement cow price (€) | **1.00** | **1.00** | **1.00** | **1.00** | **1.00** | **1.00** | **1.00** | **1.00** | **1.00** | **1.00** | **1.00** | **1.00** |
| Growth in herd size Mean | Ref. | Ref. | Ref. | Ref. | Ref. | Ref. | Ref. | Ref. | Ref. | Ref. | Ref. | Ref. |
| 10% least growth | 0.96 | 0.98 | **1.03** | 1.02 | 1.01 | 1.00 | 0.99 | 0.97 | 0.99 | **0.96** | 1.04 | **1.01** |
| 40% less growth | 0.97 | 1.01 | **0.98** | 1.00 | 0.99 | 1.00 | 0.98 | 1.01 | 1.00 | 0.99 | 1.00 | 1.01 |
| 40% more growth | 1.01 | 1.00 | **0.98** | 0.98 | 1.00 | 0.99 | 1.00 | 1.02 | 1.00 | 0.99 | 0.99 | 1.00 |
| 10% most growth | **1.07** | 1.00 | 1.00 | 0.99 | 1.00 | 1.01 | 1.02 | 1.00 | 1.02 | **1.06** | 0.97 | **0.98** |
| Annual replacement rate Mean | Ref. | Ref. | Ref. | Ref. | Ref. | Ref. | Ref. | Ref. | Ref. | Ref. | Ref. | Ref. |
| 10% least replacement | 0.94 | 0.97 | **0.94** | **0.94** | **1.05** | 1.01 | **0.93** | 0.98 | **0.92** | **0.91** | 1.03 | **1.04** |
| 40% less replacement | 0.96 | 0.99 | 1.01 | 1.00 | 1.01 | 0.99 | 0.98 | 0.99 | **0.97** | **0.95** | 1.00 | **1.01** |
| 40% more replacement | 1.02 | 1.00 | **1.03** | 1.02 | 0.98 | 0.99 | **1.06** | 1.00 | **1.03** | **1.03** | 0.98 | **0.99** |
| 10% most replacement | **1.08** | **1.04** | **1.03** | 1.05 | 0.97 | 1.02 | **1.05** | **1.04** | **1.09** | **1.12** | 0.99 | **0.97** |
| Season Mean | Ref. | Ref. | Ref. | Ref. | Ref. | Ref. | Ref. | Ref. | Ref. | Ref. | Ref. | Ref. |
| Winter (Jan-Mar) | **0.93** | **0.93** | 0.99 | 0.97 | **1.07** | **0.98** | 0.98 | **1.02** | **0.99** | **0.94** | **1.06** | 1.00 |
| Spring (Apr-Jun) | 0.98 | **0.91** | **0.94** | **0.98** | 0.99 | **1.10** | 0.96 | **0.90** | 1.00 | **0.96** | **0.94** | **1.06** |
| Summer (Jul-Sep) | **1.05** | **1.13** | **0.98** | **1.06** | **0.90** | 1.01 | 1.02 | **0.91** | **1.06** | **1.08** | **0.93** | 1.01 |
| Autumn (Oct-Dec) | 1.04 | **1.04** | **1.11** | 0.99 | **1.04** | **0.93** | 1.03 | **1.19** | **0.95** | 1.02 | **1.07** | **0.94** |
| Milk production level at herd level (mean yearly net revenue; € per cow) Mean | Ref. | Ref. | Ref. | Ref. | Ref. | Ref. | Ref. | Ref. | Ref. | Ref. | Ref. | Ref. |
| 10% lowest | 0.96 | **0.92** | 1.00 | **0.84** | **1.27** | **0.95** | **0.85** | 0.99 | **0.91** | 0.97 | 0.98 | 1.01 |
| 40% lower | **1.07** | 1.02 | **1.04** | 0.99 | **1.06** | 1.00 | 1.00 | 1.01 | 1.01 | 1.00 | **1.04** | **0.99** |
| 40% higher | 1.03 | **1.03** | 1.02 | **1.08** | **0.88** | **1.02** | **1.07** | 1.01 | **1.05** | 1.01 | 1.02 | **0.99** |
| 10% highest | 0.96 | **1.04** | 1.00 | **1.21** | **0.81** | **1.05** | **1.16** | **0.97** | 1.03 | 1.03 | 0.98 | 1.00 |
| missing | 0.99 | 0.99 | **0.94** | **0.92** | 1.05 | 0.98 | **0.95** | 1.01 | 1.01 | 0.99 | 0.98 | 1.02 |
| Herd size (mean number of cattle >2 years of age) Mean | Ref. | Ref. | Ref. | Ref. | Ref. | Ref. | Ref. | Ref. | Ref. | Ref. | Ref. | Ref. |
| 10% smallest herds | **0.74** | **0.91** | **0.80** | 0.98 | 1.01 | 0.97 | **0.81** | **0.95** | **0.92** | **0.93** | 0.98 | **1.08** |
| 40% smaller herds | 0.98 | **1.03** | **0.98** | 0.99 | 1.03 | 1.01 | 0.98 | 1.02 | 0.98 | **0.95** | 0.99 | 1.00 |
| 10% larger herds | **1.08** | **1.03** | **1.07** | 0.99 | 1.01 | 1.01 | **1.05** | 1.00 | **1.03** | 1.00 | 0.98 | **0.98** |
| 10% largest herds | **1.26** | **1.04** | **1.20** | 1.04 | **0.95** | 1.02 | **1.20** | 1.03 | **1.09** | **1.12** | 1.04 | **0.94** |
| Location of herd (province) Mean | Ref. | Ref. | Ref. | Ref. | Ref. | Ref. | Ref. | Ref. | Ref. | Ref. | Ref. | Ref. |
| Drenthe | **0.79** | **0.92** | 0.98 | 1.06 | **0.70** | 0.96 | **0.83** | 0.96 | 1.00 | 1.00 | 1.03 | **1.06** |
| Flevoland | 1.04 | 1.00 | **1.09** | 1.01 | **0.88** | 1.02 | 1.01 | 0.97 | 1.01 | 1.05 | **1.14** | 1.01 |
| Friesland | **0.46** | **1.18** | **0.90** | 1.02 | **0.88** | 1.01 | 0.95 | 0.97 | **0.78** | **0.87** | **0.82** | **1.07** |
| Gelderland | 0.96 | 1.01 | 1.00 | 0.97 | 0.98 | **1.03** | **0.96** | 0.99 | **0.95** | 0.98 | 1.01 | **1.02** |
| Groningen | **0.66** | **1.06** | **0.89** | 0.98 | **0.72** | 1.02 | 1.03 | **0.93** | **0.91** | 0.95 | **0.88** | **1.06** |
| Limburg | **1.17** | **0.91** | **1.06** | 0.99 | **0.83** | 1.02 | 1.02 | 1.02 | **1.22** | 1.04 | 1.04 | 0.99 |
| N-Brabant | **1.38** | **0.93** | **1.03** | 0.99 | **0.67** | 1.01 | **1.09** | **1.04** | **1.27** | **1.15** | **1.08** | **0.98** |
| N-Holland | **1.26** | **1.11** | 1.00 | 0.97 | **1.97** | **0.95** | **1.08** | 1.02 | 0.95 | 0.95 | 0.90 | **0.95** |
| Overijssel | **0.80** | 1.00 | **0.95** | 0.99 | **0.73** | 1.03 | **0.90** | 0.97 | **0.92** | **0.94** | **0.93** | **1.06** |
| Utrecht | **1.50** | 0.98 | **1.08** | 1.06 | **1.70** | 1.00 | 1.04 | 1.03 | **1.05** | 1.02 | 1.02 | **0.93** |
| Z-Holland | **1.37** | **0.95** | 0.99 | **0.91** | **2.27** | 1.00 | 1.04 | 1.01 | **0.95** | 1.02 | 0.99 | **0.92** |
| Zeeland | **1.23** | 0.98 | 1.05 | 1.07 | **0.86** | 0.95 | 1.08 | **1.11** | 1.07 | 1.05 | **1.23** | 0.98 |
